# A healthy dietary pattern is associated with microbiome diversity in bipolar patients: the Bipolar Netherlands Cohort (BINCO) study

**DOI:** 10.1192/j.eurpsy.2023.1201

**Published:** 2023-07-19

**Authors:** M. A. Riedinger, R. Mesbah, M. Koenders, M. Geleijnse, M. de Leeuw, N. van der Wee, E. Giltay

**Affiliations:** 1 Psychiatry, Leiden University Medical Center; 2 Psychiatry and mental disability, GGZ Rivierduinen, Leiden; 3 Bipolar disorders outpatient clinic, PsyQ, Rotterdam; 4 Psychology, Leiden University; 5 Outpatient Clinic, PsyQ, Leiden; 6 Human Nutrition & Health, Wageningen University, Wageningen; 7 Bipolar disorders, GGZ Rivierduinen, Leiden, Netherlands; 8 Biomedical Sciences, University of Antwerp, Antwerp; 9 VZW Emmaüs, University Psychiatric Hospital Duffel, Duffel, Belgium

## Abstract

**Introduction:**

The gut microbiome is one of our most prominent surfaces interacting with the outside world through the food we eat. It is influenced in terms of composition and diversity by our diets and life style habits and, in turn, affects us through the ‘gut-brain axis’. Cardiovascular risk, which is one of the main causes of death in Bipolar Disorder (BD), is affected by diet. The association between diet and microbiome in BD patients has not been studied.

**Objectives:**

We aimed to assess whether [1] dietary quality is associated with the microbiome’s diversity, and [2] what changes and interactions occur during in both the dietary quality and microbiome diversity during the subsequent year of onset BD.

**Methods:**

39 recently diagnosed patients with BD of the ‘Bipolair Nederlands Cohort’ (BINCO) (mean age 36 years, 61.5% female) were included. Food Frequency Questionnaires (FFQ) and corresponding Dutch Healthy index (DHD-15) were analyzed at baseline and one year follow-up. Feces samples corresponding to the FFQ were analyzed using 16S rDNA gene amplicon sequencing to attain the Shannon Diversity index and the Chao1 diversity index. Multivariate regression analyses were performed.

**Results:**

The Shannon diversity index significantly correlated to the DHD-15 total score after adjusting for sex and age (beta = 0.451; P = 0.004). The Chao1 index showed the same trend, but did not reach significance (beta = 0.264; P = 0.11). These positive correlations seemed to be driven by the positive effect of fish, beans, coffee, fruits and nuts. There was neither a significant change in DHD-15 index nor in the diversity measures after one year.

**Image:**

**Figure:**
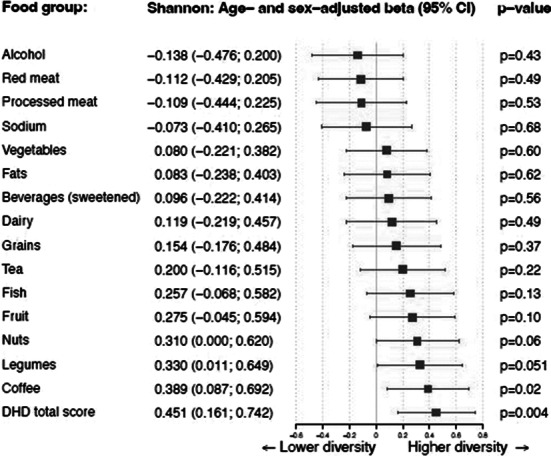

**Image 2:**

**Figure:**
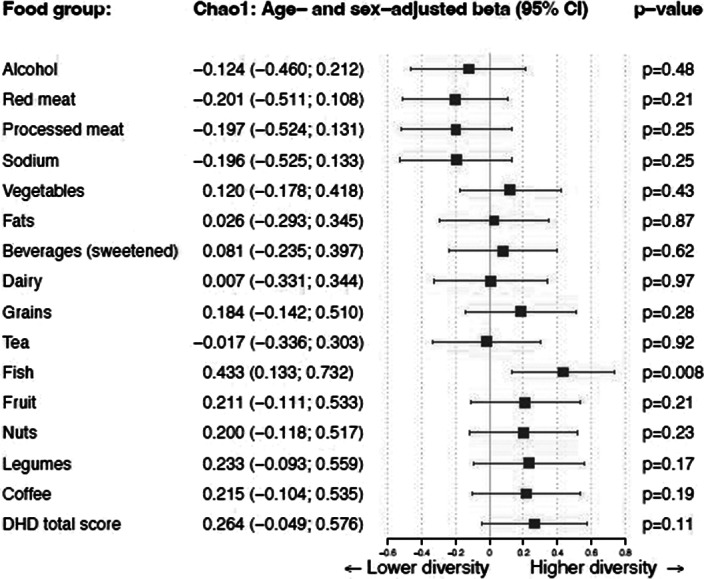

**Conclusions:**

Diversity of the microbiome is positively associated with a healthy and varied diet in BD patients, which could have consequences on mood episodes and cardiovascular risk.

**Disclosure of Interest:**

None Declared

